# Division of the Stricture in Hernia

**Published:** 1847-02

**Authors:** 


### SELECTIONS

FROM

### AMERICAN AND FOREIGN JOURNALS.

---

*Division of the Stricture in Hernia.*—The following remarks are made by Mr. Brown: It was remarked that in the above case the division of the stricture was directly upwards, which is according to the rule of the schools; it may not, however, be irrelevant to add, that in all cases of abdominal hernia,—ventral, umbilical, inguinal, and *femoral*,—I always divide the stricture directly upwards. This mode of dividing the stricture in femoral hernia is not according to the doctrine generally taught. In inguinal hernia, the seat of stricture is always the neck of the sac, formed either by the internal ring only, or, as in old hernia, by both the internal and external ring. The division of the stricture, therefore, in all cases directly upwards, will be sure to avoid the epigastric artery. In femoral hernia, we are usually taught to divide the stricture upwards and inwards, or directly inwards, to cut Gimbernat's ligament; but by the adoption of this mode, if the obturator artery should chance to rise from the epigastric, and take its course along the margin of Gimbernat's ligament, it might be wounded by the most skilful operator. I once knew this to occur, and as the hemorrhage was excessive, the surgeon with great promptness tied the external iliac artery, which immediately arrested it. Besides, the true seat of stricture in femoral hernia is not in Gimbernat's ligament, but in the "thin fibrous band which crosses the vessels beneath Poupart's ligament." By cutting this "thin fibrous band" directly upwards, "the ring becomes instantly flaccid," and the hernia is readily reduced.—*Lond. Lancet.*

